# Assessment of volume flow rate in arteriovenous fistulas with a novel ultrasound Doppler device (earlybird): Trend analysis, comparison of methods, and inter- and intra-rater reliability

**DOI:** 10.1177/11297298241250379

**Published:** 2024-05-06

**Authors:** Emilie Holst-Jæger, Marthe Barstad, Øyvind Salvesen, Hans Torp, Arne Seternes, Erik Mulder Pettersen

**Affiliations:** 1 Faculty of Medicine and Health Sciences, Norwegian University of Science and Technology, Trondheim, Norway; 2 Clinical Research Unit, Department of Clinical and Molecular Medicine, Norwegian University of Science and Technology, Trondheim, Norway; 3 Department of Circulation and Medical Imaging, Norwegian University of Science and Technology, Trondheim, Norway; 4 CIMON Medical, NTNU Technology Transfer AS, Trondheim, Norway; 5 Section of Vascular Surgery, Department of Surgery, Trondheim University Hospital, St. Olavs Hospital, Trondheim, Norway; 6 Research Center for Medical Equipment, Technology and Innovation, St. Olavs Hospital, Trondheim, Norway; 7 Department of Surgery, Sørlandet Hospital Kristiansand, Kristiansand, Norway

**Keywords:** Vascular access, arteriovenous fistula, hemodialysis, surveillance, volume flow rate, ultrasonography, duplex, Doppler, earlybird, reliability, kidney failure, renal disease

## Abstract

**Background:**

An accessible tool is required to analyze volume flow trends in arteriovenous fistulas for hemodialysis. Earlybird, an easy-to-place ultrasound Doppler device, has shown comparable accuracy to duplex ultrasound. In this study, we compared volume flow measurements obtained with duplex ultrasound and the dilution technique to an enhanced earlybird device, featuring a dual Doppler probe system, eliminating the requirement for a known insonation angle.

**Methods:**

Nine patients with a distal radiocephalic arteriovenous fistula were monitored for 12 months with regular volume flow measurements. Correlation and inter- and intra-class reliability analyses were conducted.

**Results:**

An overall moderate correlation was observed between earlybird and duplex ultrasound or dilution technique (intraclass correlation coefficient = 0.606 (95% confidence interval 0.064, 0.721) and 0.581 (0.039, 0.739), respectively). Duplex ultrasound compared to dilution measurements, demonstrated an overall moderate correlation (0.725 (0.219, 0.843)). Correlation between earlybird and duplex ultrasound was stronger for the arteriovenous fistula (0.778 (0.016, 0.901)) than the brachial artery (0.381 (−0.062, 0.461)). For earlybird, inter-rater reliability was excellent for the arteriovenous fistula (0.907 (0.423, 0.930)) and poor for the brachial artery (0.430 (0.241, 0.716)). Duplex ultrasound showed a good inter-rater reliability (arteriovenous fistula: 0.843 (0.610, 0.871), brachial artery: 0.819 (0.477, 0.864)). The overall intra-rater reliability was good for duplex ultrasound (rater A: 0.893 (0.727, 0.911); rater B: 0.853 (0.710, 0.891)), while excellent for earlybird (rater A: 0.905 (0.819, 0.928); rater B: 0.921 (0.632, 0.969)).

**Conclusion:**

We observed a weaker correlation in the measurements of volume flow rates in arteriovenous fistulas when obtained using earlybird compared to dilution technique, unlike the comparison between duplex ultrasound and the dilution technique. However, inter-rater reliability for the arteriovenous fistula was excellent with earlybird and good with duplex ultrasound, indicating the potential of earlybird as a tool for frequent measurements, enabling trend surveillance and predicting adverse outcomes.

## Introduction

Ensuring a reliable and durable vascular access is crucial for maintaining the quality of dialysis in patients with end-stage renal disease.^1^ Arteriovenous fistula is preferred due to higher patency, lower infection, and overall lower morbidity.^1,2^ Challenges exist regarding fistula maturation and functionality, with varying guidelines on surveillance utility.

National Kidney Foundation Kidney Disease Outcomes Quality Initiative (KDOQI) recommends regular clinical examination for arteriovenous fistula dysfunction.^3^ Monitoring volume flow rate and trend surveillance might improve fistula patency.^2^ European Renal Best Practise and KDOQI conclude that the evidence is inadequate to make a recommendation on routine arteriovenous fistula surveillance at regular intervals.^3,4^ European Society for Vascular Surgery (ESVS) recommends surveillance every 3 months through flow measurements. However, ESVS acknowledges that the rationale for vascular access surveillance programs may be flawed, and suggests that trend analysis might offer better guidance for decisions on referrals compared to relying on a single measurement.^5^ A reliable, cost-effective, user-friendly method is necessary for frequent assessment.^5^

Duplex ultrasound concurs with the hemodilution technique, with lower cost and technical failure rate.^6–9^ Magnetic resonance angiography is a viable option, albeit costly and extensively employed.^5^ However, all volume flow rate measurements have low reproducibility.^5,10,11^

Our research group proposes using trend analysis with earlybird, a Doppler ultrasound monitoring device, for surveillance of volume flow rate measurements. Earlybird incorporates a highly sensitive ultrasound Doppler probe with a large surface area, facilitating convenient positioning and enabling uninterrupted assessments of peripheral circulation. Compared to duplex ultrasound, earlybird with a single probe demonstrated higher accuracy in measuring volume flow rate.^6^ To address angular correction limitations, a dual probe system was incorporated.^12,13^ A customized software analyses Doppler spectrograms to calculate angle-corrected peak systolic velocity and diameter.

The aim of this study was to compare earlybird dual Doppler probe system with ultrasound and hemodilution technique for measuring volume flow rate in patients with arteriovenous fistulas for hemodialysis, and to assess the intra- and inter-rater reliability of duplex ultrasound and earlybird. Additionally, this study explores the feasibility of earlybird as a tool for trend analysis.

## Patients and method

The present study is a prospective feasibility study to compare earlybird with dual probe against duplex ultrasound and dilution technique to calculate volume flow rate, and thereby assess its suitability for trend analysis for surveillance of arteriovenous fistulas for hemodialysis.

### Patients

Nine patients were recruited from the dialysis unit at St. Olavs Hospital, and volume flow rate measurements were performed between January 2022 and December 2022. Inclusion criteria were an established and mature distal radiocephalic arteriovenous fistula. The only exclusion criterion was non-competence to give consent. Two patients did not complete the 12-months follow-up, due to kidney transplantation and personal circumstances, respectively. The other seven were followed up with 7–9 measurements at 6–8 weeks intervals. The patients were non-fasting, and all measurements were done prior to cannulation of the fistula. Arteriovenous fistula flow dysfunctions and eventually interventions were registered. Health information for each patient was obtained from medical records.

### Measurements

Systolic and diastolic blood pressure and weight measured prior to cannulation, and dilution measurements performed every 3 months, were obtained from the Therapy Data Management System. All dilution measurements were conducted by dialysis nurses. Inexperienced examinators performed earlybird and duplex ultrasound volume flow rate measurements after receiving training from two experienced examiners. Volume flow rate measurements were performed on the arteriovenous fistula outflow vein and the ipsilateral brachial artery. Earlybird and duplex ultrasound measurements were repeated twice at each location by both examiners, resulting in a total of eight duplex ultrasound and eight earlybird measurements per patient visit. Notably, dilution flow measurements were not conducted during the same visit as the other flow measurements. The probe was repositioned for each measurement, and it was assumed that the volume flow rate was unchanged during the short duration of measurements.

#### Duplex ultrasound

Duplex ultrasound measurements were conducted using the GE Vivid E95 (General Electric, Vingmed Ultrasound, Horten, Norway) with a 9L linear transducer. The vessel of interest was visualized at the optimal location for volume flow rate measurements, considering anatomical variations and aiming to achieve laminar flow. Stable volume flow curves were obtained. Angle-corrected pulse wave Doppler with an insonation angle below 60° was employed. Volume flow rate was automatically calculated based on the diameter and the intensity-weighted mean frequency of one heart cycle.

#### Earlybird

**Figure 1.**
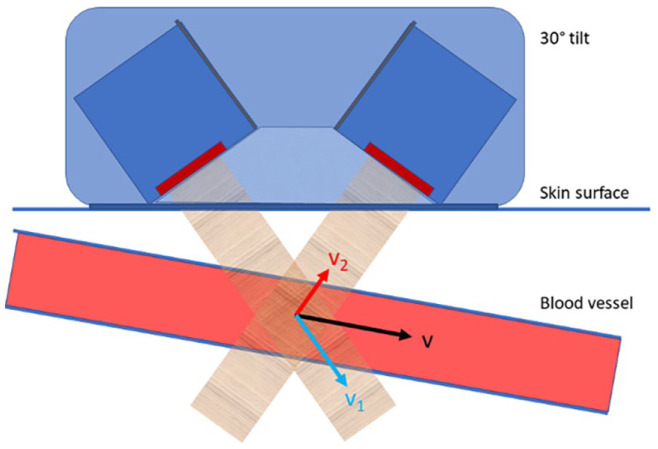
The earlybird dual-probe system consisted of two high-sensitive pulsed Doppler ultrasound probes. These probes were tilted 30° giving a fixed insonation angle of 60° to the skin surface, in a fixed 3D printed case, ensuring a consistent and known angle between them. A vector model-based algorithm calculated the peak systolic velocity (v) and diameter based on the respective velocities measured by the two probes (v1 and v2) and used to determine volume flow rate.

Earlybird is an ultrasound Doppler monitoring device that is equipped with two transducers, acquisition hardware, and a user interface.^14^ Both probes are fixed in a case that ensures 60° insonation angle to the skin (Figure 1). The nominal frequency is 4 MHz, transmitted at a pulse repetition rate of 8 kHz. Software developed in MATLAB was used, and recordings were made with “multi-gated” Doppler signals for later processing of “Doppler-M program” and spectrogram.^6^ The vessel diameter and volume flow rate were calculated using signals from both probes, eliminating the need to determine the actual insonation angle. The probe was positioned guided by real-time Doppler spectrogram, to achieve laminar flow and to make sure that both probes received adequate signal (Figure 1).

### Hemodilution technique

Hemodilution measurements were conducted using HD03 Hemodialysis Monitor (Transonic Systems Inc, Ithaca, USA). Both venous and arterial needles were inserted in the direction of blood flow (antegrade cannulation), spaced 2–10 cm apart. The flow rate in the dialysis circuit, referred to as autoflow, was individually adjusted within a reference range of 350–450 ml/min. Autoflow was determined by the autoflow factor, calculated based on the size of the dialysis filter. Notably, hemodialysis filtration employed a larger filter area (2.3 m^2^) compared to standard hemodialysis (1.4 m^2^), resulting in an increased flow rate in the dialysis circuit.

### Statistical analysis

We excluded data samples that were extracted from a faulty measurement, which could be based on measurement on a different vessel than the one of interest. The samples were excluded on peak systolic velocity and diameter calculations as well as the pulsatile and resistance index. The data was arranged in pairs with all possible combinations of measurements conducted on the same study subject on the same visit. The nine IDs were bootstrapped to find the confidence interval for intraclass correlation coefficient (ICC). Correlation among duplex ultrasound, dilution technique, and earlybird were investigated, and Pearson’s correlation applied to the arranged pairs gave the ICC. Some analyses were conducted considering the same rater and/or localization, while others were performed independent of these assumptions. ICC below 0.50 was considered poor; 0.50–0.75, moderate; 0.75–0.90, good; and above 0.90, excellent.^15^ All ICCs are presented with a 95% confidence interval. Bland-Altman plots were calculated based on the relative difference of the measured volume flow rate, by each method, from the mean volume flow rate per patient per visit derived from combining measurements from earlybird, duplex ultrasound, and the hemodilution technique.^16,17^ Consistent bias expressed as the mean relative difference was assessed using One-Sample *t*-test. Linear regression was used to assess proportional bias. Continuous data are presented with median and percentiles. Baseline data is presented in numbers. SPSS (IBM Corp. IBM SPSS Statistics for Windows, Version 29.0. Armonk, NY) and R (Version 3.63) was used for statistical analysis. MySQL was used to create pairs.

### Ethics

The Regional Committee for Medical and Health Research Ethics in Central Norway has approved this study (044/2017). All participants provided written informed consent.

## Results

In total, nine patients (eight males; mean age 61 (32–79) years; mean BMI 29.4 (23.8–34.9) kg/m^2^) were included in the present study. Median time from AV-fistula creation to study start was 21.7 months (range 16.7–55.9). Median baseline flow for hemodilution measurements was 700 ml/min (range 180–1950). Three patients (IDs 1, 4, and 8) had undergone prior interventions, each undergoing percutaneous transluminal angioplasty procedures ranging from four to seven times per patient. The last access-related intervention was performed 6 months, 2 weeks, and 1 month, respectively, before the study commenced. None were active smokers, and six quit smoking more than 10 years ago. Two patients had atrial flutter.

Out of a total of 986 volume flow rate measurements, 58 measurements was conducted using the hemodilution technique, 464 with earlybird, and 464 with duplex ultrasound. 821 were eligible for further analysis, while 165 were non-eligible measurements. The absence of one rater led to 16 missing values, including eight earlybird measurements and eight duplex ultrasound measurements. Additionally, technical errors associated with the earlybird system accounted for another eight missing values. In 19 measurements on the brachial artery, and six measurements on the arteriovenous fistula outflow vein, volume flow calculation was not feasible with the earlybird system. A total number of 110 earlybird volume flow measurements were excluded due to predefined criteria, including 58 measurements conducted on the brachial artery and 52 measurements conducted on the arteriovenous fistula. Correspondingly, a total of five duplex ultrasound measurements were excluded due to predefined criteria, including one measurement conducted on the arteriovenous fistula and five measurements conducted on the brachial artery. Lastly, one value was not saved and therefor missing for the earlybird measurement of volume flow of the arteriovenous fistula outflow vein. In total, 33% of earlybird measurements could not be used in the analyses, compared to 3% of duplex ultrasound measurements.

### Comparison of methods (earlybird, duplex ultrasound, and hemodilution technique)

An overall moderate correlation was observed between earlybird and duplex ultrasound (ICC = 0.606 (95% confidence interval 0.0644, 0.721)), as well as between earlybird and dilution measurements (ICC = 0.581 (0.039, 0.739)). Duplex ultrasound correlated to dilution measurements also demonstrated an overall moderate correlation (ICC = 0.725 (0.219, 0.843)).

**Figure 2.**
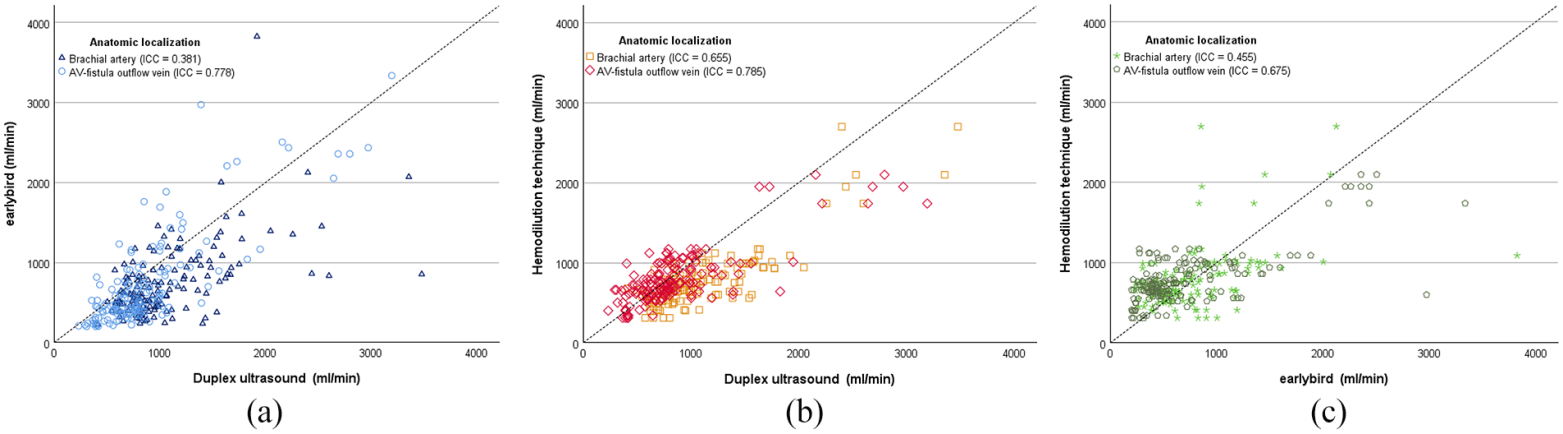
Scatter plot of correlation of volume flow rate (ml/min) of (a) duplex ultrasound (*x*-axis) and earlybird (*y*-axis), (b) duplex ultrasound (*x*-axis) and hemodilution technique (*y*-axis), and (c) earlybird (*x*-axis) and hemodilution technique (*y*-axis). The anatomic localizations for the measurements, specifically the brachial artery and arteriovenous fistula (AV-fistula outflow vein), are marked separately. The identity line is given. Correlations are given as intraclass correlation coefficient (ICC).

A consistent pattern was observed across modalities, indicating a stronger correlation for measurements of the arteriovenous fistula outflow vein compared to those of the brachial artery (Figure 2). For earlybird compared to duplex ultrasound, there was a poor correlation for the brachial artery (ICC = 0.381 (−0.062, 0.461)) but a good correlation for the arteriovenous fistula outflow vein (ICC = 0.778 (0.016, 0.901)). Duplex ultrasound exhibited a moderate to good correlation with the hemodilution technique, measured at the brachial artery (ICC = 0.655 (0.094, 0.841)) and the arteriovenous fistula outflow vein (ICC = 0.785 (0.267, 0.845)). A comparable trend was observed when comparing earlybird with the hemodilution technique, showing a stronger correlation for earlybird volume flow rate measured at the arteriovenous fistula outflow vein (ICC = 0.675 (−0.059, 0.828)) than for the earlybird measurements made at the brachial artery (ICC = 0.455 (0.068, 0.606)).

### Analysis of variance (Bland-Altman plots)

The relative difference of earlybird from the mean volume flow rate per patient-visit showed a significant constant bias for both the volume flow rate measured at the brachial artery (mean = −9.04% (SE 2.98), *p* = 0.136, with 95% limits of agreement – 79.9 and 61.9) and for the AV-fistula outflow vein (mean = −24.7% (SE 1.92), *p* < 0.001, with 95% limits of agreement −73.0 and 23.6). The means of the relative difference were significantly different (*p* < 0.001). There was no proportional bias for earlybird measured at the brachial artery (*B* = 0.011 (SE 0.007), *p* = 0.136), but a significant proportional bias when measured at the AV-fistula outflow vein (*B* = 0.026 (SE 0.004), *p* < 0.001).

**Figure 3.**
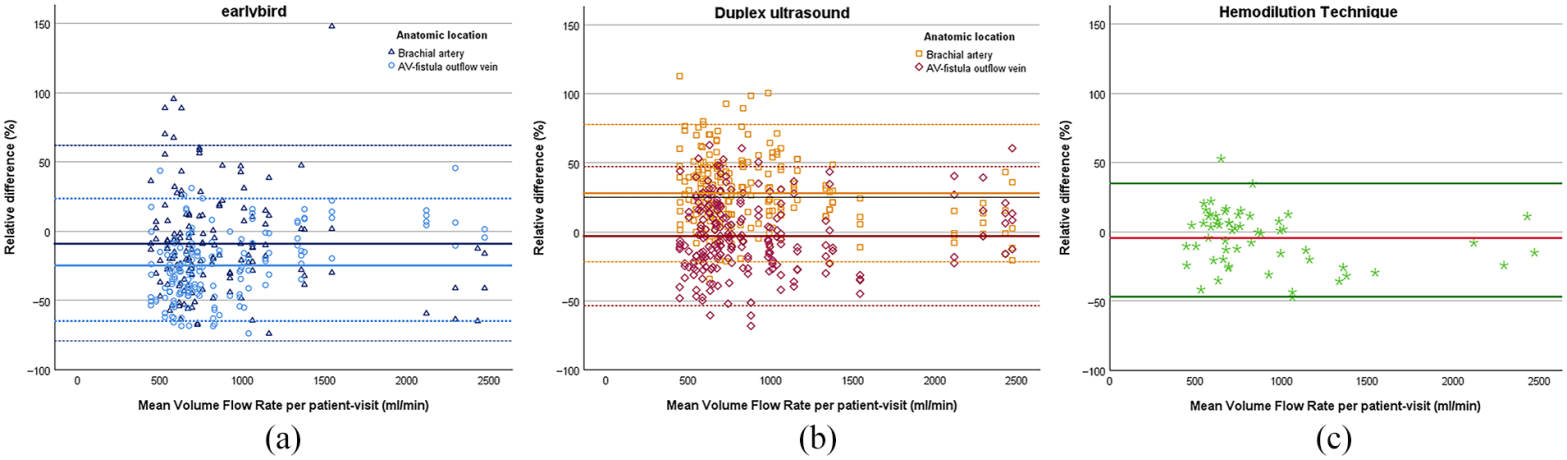
Bland-Altman plots of the relative difference (%) of earlybird (a), Duplex Ultrasound (b), and hemodilution technique (c), respectively, and the mean volume flow rate per patient-visit (ml/min), plotted for anatomic location separately. Mean relative difference (solid line) and 95% limits of agreement (dotted line) are given.

The relative difference of Duplex ultrasound from the mean volume flow rate per patient-visit showed a constant bias for the volume flow rate measured at the brachial artery (mean = 28.0% (SE 1.70), *p* < 0.001, with 95% limits of agreement – 21.7 and 77.8) but not for the AV-fistula outflow vein (mean = −3.13% (SE 1.71), *p* = 0.068, with 95% limits of agreement −53.5 and 47.2). The means of the relative difference were significantly different (*p* < 0.001). There was a proportional bias for Duplex ultrasound measured at the brachial artery (*B* = −0.014 (SE 0.004), *p* < 0.001) but there was not a proportional bias when measured at the AV-fistula outflow vein (*B* = 0.004 (SE 0.004), *p* = 0.242) (Figure 3).

### Inter- and intra-reliability analyses

**Figure 4.**
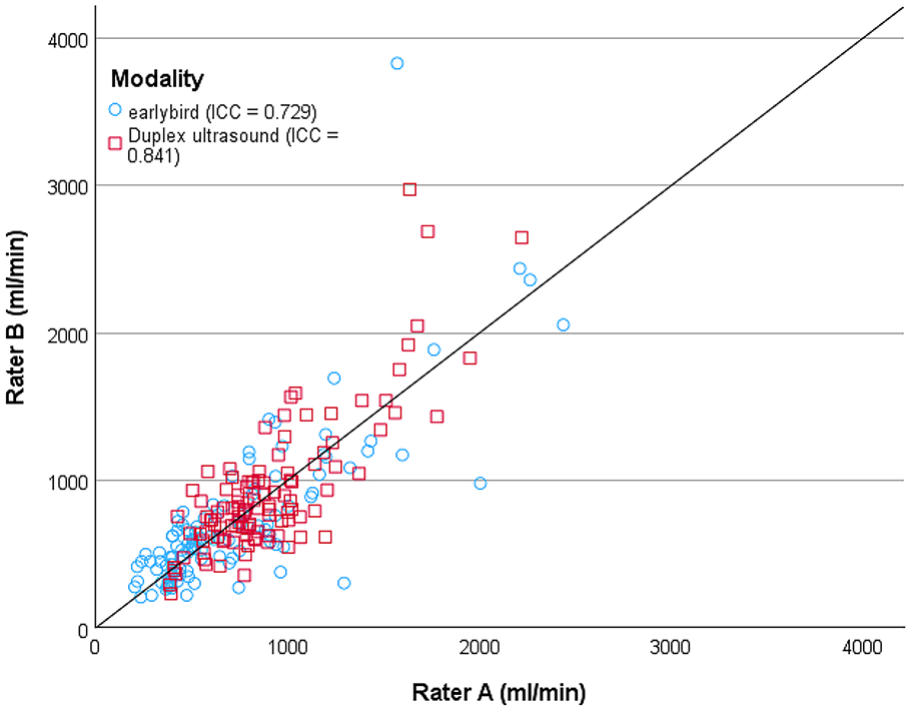
Scatter plot of inter-rater reliability of volume flow rate (ml/min) measured with duplex ultrasound and earlybird with rater A at *x*-axis and rater B at *y*-axis. The identity line is given. Correlations are given as intraclass correlation coefficient (ICC).

A similar overall good inter-rater reliability was found for both earlybird and duplex ultrasound, however it seems that ultrasound is more eligible for measuring volume flow at the brachial artery, while earlybird performs excellent at measuring volume flow at the outflow vein of the AV-fistula. An overall moderate inter-rater reliability was found for earlybird (ICC = 0.729 (0.548, 0.873)) and good for duplex ultrasound (ICC = 0.841 (0.641, 0.867)) (Figure 4). For the measurements made at arteriovenous fistula outflow vein, the inter-rater reliability was excellent for earlybird (ICC = 0.907 (0.423, 0.930)) and good for duplex ultrasound (ICC = 0.843 (0.610, 0.871)). For the brachial artery, the inter-rater reliability was poor for earlybird (ICC = 0.430 (0.241, 0.716)) and good for duplex ultrasound (ICC = 0.819 (0.477, 0.864)) (see Figure 4).

**Figure 5.**
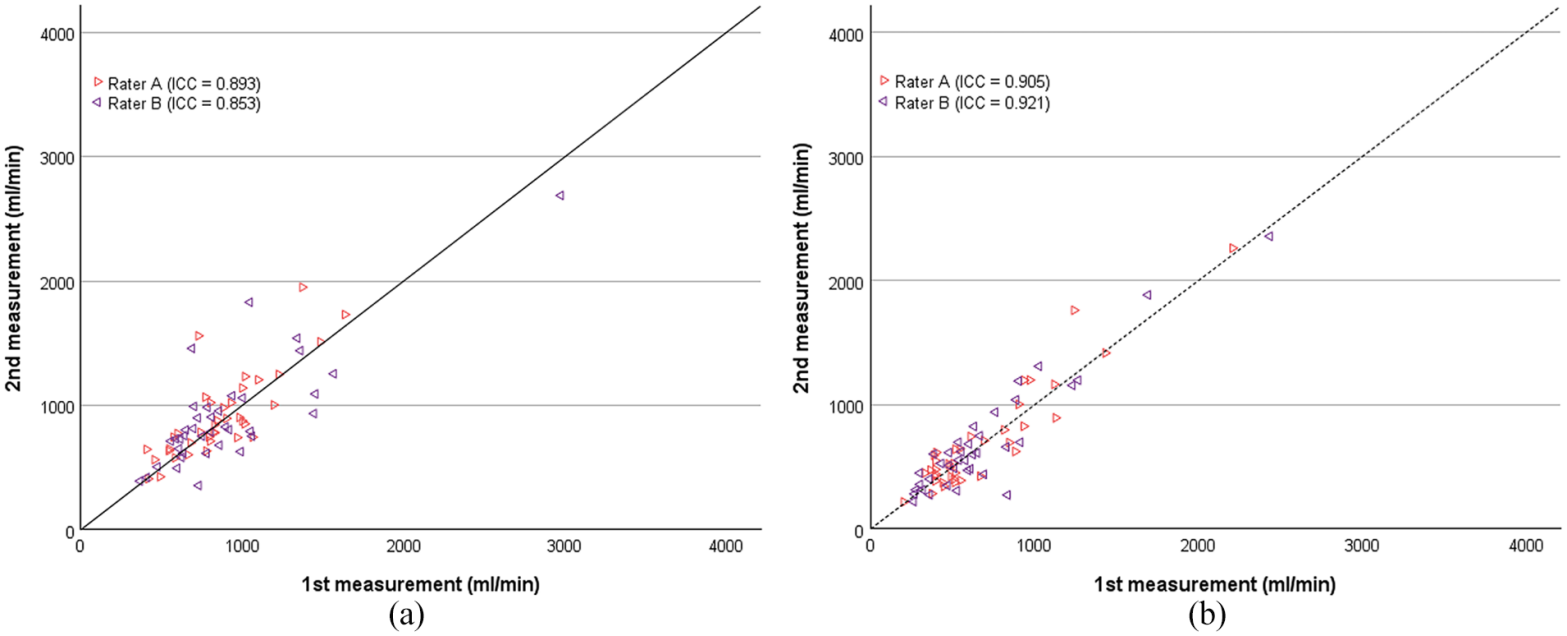
Scatter plot of intra-rater reliability of volume flow rate measurements (ml/min) with (a) duplex ultrasound and (b) earlybird. Rater A and B are marked separately. First measurement conducted with the respective devices are plotted at the *x*-axis, second measurement at the *y*-axis. The identity line is given. Intra-rater reliabilities are given as intraclass correlation coefficient (ICC).

The overall intra-rater reliability was good for duplex ultrasound, both for rater A (ICC = 0.893 (0.727, 0.911)) and rater B (ICC = 0.853 (0.710, 0.891)). Earlybird showed comparable results, with an overall intra-rater reliability which was excellent, both for rater A (ICC = 0.905 (0.819, 0.928)), and for rater B (ICC = 0.921 (0.632, 0.969)) (Figure 5).

When measuring at arteriovenous fistula outflow vein, rater A demonstrated an excellent intra-rater reliability for earlybird (ICC = 0.931 (0.659, 0.943)) and a good intra-rater reliability for duplex ultrasound (ICC = 0.873 (0.671, 0.904)). Similarly, rater B showed excellent intra-rater reliability for earlybird (ICC = 0.948 (0.429, 0.984)) and good intra-rater reliability for duplex ultrasound (ICC = 0.862 (0.449, 0.928)). For measurements taken at the brachial artery, duplex ultrasound exhibited stronger intra-rater reliability for both raters: rater A (ICC = 0.909 (0.588, 0.935)); rater B (ICC = 0.818 (0.677, 0.856)), compared to earlybird: rater A (ICC = 0.827 (0.321, 0.740)); rater B (ICC = 0.786 (0.469, 0.881)).

### Trend analysis

**Figure 6.**
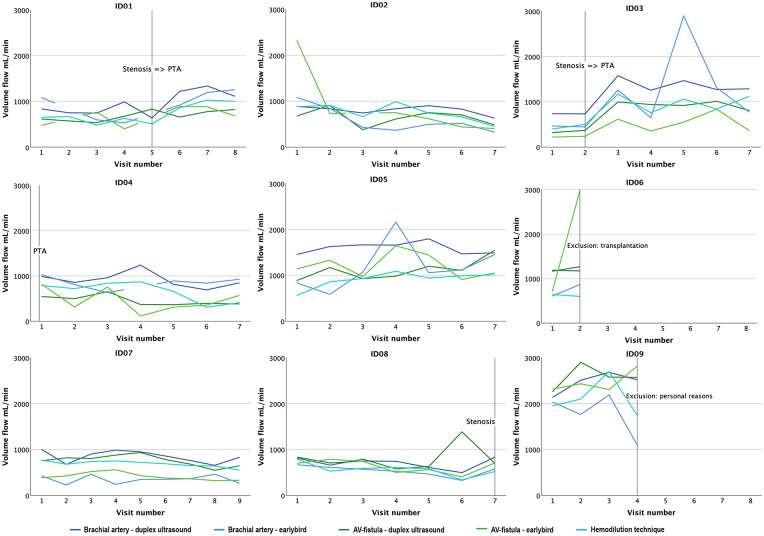
Trend charts for each patient. Volume flow rates (ml/min) are given in mean values for each visit, location; arteriovenous fistula (AV-fistula) and brachial artery, and modality; earlybird, duplex ultrasound, and hemodilution technique. Adverse events, eventual treatment, and loss of follow-up are marked with a vertical line and described.

Individual trend charts, presented as the average volume flow rate (ml/min), emphasize the consistent pattern across modalities, revealing a visually altered volume flow rate in the two patients who underwent percutaneous transluminal angioplasty (PTA) for stenosis treatment within the follow-up period (Figure 6).

## Discussion

There was an overall stronger correlation between duplex ultrasound and hemodilution technique, compared to the correlation between earlybird and the two other modalities. These findings align with the prevailing use of duplex ultrasound as a the preferred method for assessing fistula maturing and functioning.^6–9^ Nevertheless, this is in contrast to the findings of Zanen et al., who observed a correlation coefficient of *r* = 0.35 and ICC = 0.1 when comparing the hemodilution technique and duplex ultrasound.^18^ Conversely, Depner and Krivitski reported a correlation coefficient of *r* = 0.69.^19^ Our study, however, revealed that the hemodilution technique displayed narrower 95% limits of agreement in the Bland-Altman plots and correlated more effectively with earlybird and duplex ultrasound.

Duplex ultrasound demonstrated good to excellent inter- and intra-rater reliability, whereas earlybird exhibited poor to excellent reliability, especially depending on the anatomical position of the measurement. Variations in intra- or inter-rater reliability analyses are commonly acknowledged as potential error sources in Doppler ultrasound measurements.^20,21^ However, to our knowledge, there are no existing reports on the inter- and intra-rater reliability analysis of duplex ultrasound volume flow measurements, specifically for radiocephalic arteriovenous fistulas.

The earlybird dual probe, designed for angle-independent volume flow rate measurements, requires good signals and an insonation angle of approximately 60° for accuracy (Figure 1). Optimal conditions are found in superficial vessels parallel to the skin, supporting earlybird’s suitability for arteriovenous fistula outflow vein measurements. However, deeper vessels like the brachial artery may lead to less accurate readings due to more perpendicular probe angles. Earlybird lacks anatomical imaging but provides M-mode, Doppler spectrograms, and records blood flow velocities within the specified depth. The arteriovenous fistula outflow vein, sensitive to disturbances in parabolic laminar flow, might be susceptible to suboptimal probe positioning or unintentional measurement of the wrong vessel. Such occurrences could be influenced by vessel characteristics and the examiner’s lack of experience. The high proportion of excluded samples from the earlybird measurements was attributed to probe misplacement, highlighting the necessity for targeted efforts to optimize and further develop the earlybird device. In a previous study, we found greater accuracy and less bias for volume flow measurements conducted with earlybird compared to duplex ultrasound.^6^ In this study, the examinator had a deeper understanding of earlybird and was an experienced ultrasound examiner. For further studies, volume flow measured with earlybird at the AV-fistula outflow vein by inexperienced operators, compared to conventional use of duplex ultrasound with experienced operators, might contribute to valuable findings. Incorporating visual feedback or a positional guiding mechanism for earlybird would improve the above-mentioned challenges and could allow for frequent volume flow rate measurements by non-specialized personnel and even patients themselves, potentially at each hemodialysis visit or at home.

The Bland-Altman plots indicate that the limits of agreement are far wider (>50%), for both duplex ultrasound and earlybird, than the changes in flow that would usually trigger interventions in clinical practice, which is regarded more than a 33% change.^5,22^ The above-mentioned factors, like inexperienced ultrasound examiners, improved earlybird device, and choice of anatomical positioning, may improve the volume flow calculation and could potentially contribute to narrowed limits of agreements.

Trend monitoring of volume flow rate, especially with earlybird, was evaluated in terms of assessing the ability of predicting adverse events. The lack of statistical analyses prevents drawing conclusions, but visual qualitative assessments allow for a suggestion. The trend charts visualize the low reproducibility of volume flow rates across modalities with a wide range between measurements from each visit. Compared to earlybird, duplex ultrasound seems to visually measure higher volume flow rates, as described in earlier studies.^6^ The charts also indicate that volume flow rate increases, captured by all modalities, after endovascular stenosis treatment (Figure 6) (study subject 1, 3). This finding supports the value of trend analysis of volume flow rate for early detection of complications.

### Strengths and limitations

This study has several strengths, notably the extensive number of measurements conducted on patients within a clinical setting, a comparative analysis involving three modalities, and consistency in raters performing both duplex ultrasound and earlybird measurements. Limitations of this study include a relatively small cohort of patients, infrequent measurements, a large amount of excluded measurements due to a misplaced probe or inadequate sample quality, and a limited level of ultrasound experience among examiners. Additionally, the temporal discrepancy between hemodilution measurements and other measurements may have been influenced by variations in patients’ hemodynamics.

For duplex ultrasound, a primary challenge lies in its user-dependency.^23^ Future studies may benefit from including a larger number of raters to assess variations in measurement. Additionally, both earlybird and duplex ultrasound encounter the challenge of increased risk of non-laminar flow in the arteriovenous fistula outflow vein.

## Conclusion

Earlybird equipped with a dual probe system is a feasible tool to measure volume flow AV-fistulas in patients established in hemodialysis. However, due to its technical specification, it exhibits less consistency in measuring volume flow rates at the brachial artery. As such, the arteriovenous fistula outflow vein should be the preferred anatomic location for measurements using earlybird. This preference is supported by the excellent inter- and intra-rater reliability demonstrated with earlybird.

Nevertheless, in our comparisons among methods, a stronger correlation was observed between duplex ultrasound and the hemodilution technique compared to earlybird.

The outcomes of this study offer insights into a potential targeted application of earlybird. Considering its lightweight design and its easy deployment, there is a strong prospect that with enhanced functionality, earlybird could emerge as a tool suitable for frequent measurements, facilitating trend surveillance and the prediction of adverse outcomes.
